# Knowledge, Perceptions and Practices of Community Pharmacists Towards Antimicrobial Stewardship: A Systematic Scoping Review

**DOI:** 10.3390/antibiotics8040263

**Published:** 2019-12-12

**Authors:** Sajal K. Saha, Chris Barton, Shukla Promite, Danielle Mazza

**Affiliations:** 1 Department of General Practice, Monash University, Building 1, 270 Ferntree Gully Road, Notting Hill, VIC 3168, Australia; Chris.Barton@monash.edu (C.B.); Danielle.mazza@monash.edu (D.M.); 2 National Centre for Antimicrobial Stewardship (NCAS), The Peter Doherty Institute for Infection and Immunity, Melbourne, VIC 3168, Australia; 3 Department of Infection Immunity and Human Disease, University of Leeds, Leeds LS2 9JT, UK; bs18sp@leeds.ac.uk

**Keywords:** antimicrobial stewardship, community pharmacist, AMS survey tools, knowledge, perceptions, practices

## Abstract

The scope of antimicrobial stewardship (AMS) surveys on community pharmacists (CPs) is uncertain. This study examines the breadth and quality of AMS survey tools measuring the stewardship knowledge, perceptions and practices (KPP) of CPs and analyse survey outcomes. Following PRISMA-ScR checklist and Arksey and O’Malley’s methodological framework seven medical databases were searched. Two reviewers independently screened the literatures, assessed quality of surveys and KPP outcomes were analysed and described. Ten surveys were identified that assessed CPs’ AMS perceptions (*n* = 7) and practices (*n* = 8) but none that assessed AMS knowledge. Three survey tools had been formally validated. Most CPs perceived that AMS improved patient care (median 86.0%, IQR, 83.3–93.5%, *n* = 6), and reduced inappropriate antibiotic use (84.0%, IQR, 83–85%, *n* = 2). CPs collaborated with prescribers for infection control (54.7%, IQR 34.8–63.2%, *n* = 4) and for uncertain antibiotic treatment (77.0%, IQR 55.2–77.8%, *n* = 5). CPs educated patients (53.0%, IQR, 43.2–67.4%, *n* = 5) and screened guideline-compliance of antimicrobial prescriptions (47.5%, IQR, 25.2–58.3%, *n* = 3). Guidelines, training, interactions with prescribers, and reimbursement models were major barriers to CP-led AMS implementation. A limited number of validated survey tools are available to assess AMS perceptions and practices of CPs. AMS survey tools require further development to assess stewardship knowledge, stewardship targets, and implementation by CPs.

## 1. Introduction

Antimicrobial resistance (AMR) is a pressing concern of community health [1]. Given the majority of antimicrobials being prescribed and dispensed in the community, the use of antimicrobial stewardship (AMS) programs is essential in this setting to address AMR [2]. 

AMS aims to improve patient care and decrease health care costs associated with infections through coordinated interventions on the choice, dose, dose-regimen, side effects, drug interactions and allergies of prescribed antibiotics [3]. To facilitate these coordinated interventions of AMS, both general practitioners (GPs) and community pharmacists (CPs) have central roles to play as they are the first point of contact for patients in the community. To date, research has focused more on exploring GPs’ experiences with AMS strategies [4] compared to CPs. 

Research has shown that CP-led AMS strategies [5] are effective in optimising antibiotic use. Examples include providing patient education [6], recommending symptomatic management to patients with urinary tract infections [7] and managing pharyngitis and bronchitis patients using point-of-care test in a GP-CP collaborative model [8]. However, the roles of CPs in AMS are not either well defined or guided by the national policy globally even though the urgency of community AMS has been advocated [9]. 

The Global Respiratory Infection Partnership (GRIP) framework has included pharmacy leadership as one of the essential parts in the provision of advice to prescribers and patients [10]. CPs are well-positioned to collaborate and communicate with GPs and patients if an antimicrobial prescription is believed to be inappropriate [8,9,11]. Multiple studies [12,13,14] reported that providing recommendations on the choice, and dose of antimicrobial prescriptions and ability to assess appropriateness of GPs’ antimicrobial prescriptions by CPs depend on adequate knowledge, skills and positive attitudes of CPs towards AMS. CPs’ ability to implement AMS in collaboration with GPs has been shown to depend on the context where they work [15,16]. To date, the scope of evidence regarding the uptake of AMS strategies by CPs and barriers to apply AMS strategy during community pharmacy practice is not known.

A 2019 systematic review and meta-analysis demonstrated that GP-pharmacist collaborative AMS strategies are effective in reducing antibiotic prescribing (up to 12%) and improving guideline-adherent prescribing (up to 16%) by GPs [17]. Nevertheless, GP-CP collaborative AMS implementation models to optimise antimicrobial use in community are less explored worldwide [8,18].

AMS surveys have been widely used in hospital settings to determine the status of AMS implementation [19] and changes of pharmacists’ role and attitudes concerning AMS [20]. In contrast, the scope of AMS surveys from CPs’ perspectives is uncertain. This review aimed to determine the breadth of existing AMS surveys and tools for future use in stewardship monitoring and to measure and report the knowledge, perceptions and practices of CPs regarding AMS. 

## 2. Methods

A scoping review methodology [21,22,23] was employed to conduct this study using the checklist of PRISMA extension for scoping reviews (PRISMA-ScR) [24] (Supplementary Table S1) and a methodology framework described by Arksey and O’Malley [21] consisting of seven stages. The stages of the methodology framework undertaken were: (1) Identification of research objectives; (2) reviewing data sources and search strategies for the identification of studies; (3) study selection; (4) data extraction; (5) assessing the quality of included studies; (6) collating, summarising and analysing outcome evidence and (7) describing future direction of further research.

### 2.1. Research Objectives

This review focused on the below specific objectives.

1. To identify the breadth and scope of existing AMS surveys targeting CPs.
2. To assess the quality of and gaps in AMS survey tools measuring knowledge, perceptions and practices of CPs regarding AMS.
3. To identify and analyse the types and range of outcomes reported in AMS surveys.
4. To identify the evidence gaps and recommend future directions of research in relation to improving AMS in community pharmacy.

### 2.2. Reviewing Data Sources and Search Strategies

A systematic search was conducted in seven databases of Medline, Embase, Emcare, Pubmed, CINAHL, Web of Science, and Pharmaceutical Abstracts from their inception to 30 October 2018. Databases were accessed through the Monash University library systems. Employed search strategy was: [(antimicrobial stewardship OR antibiotic stewardship) AND (knowledge* OR attitude* OR perception* OR belief* OR practice* OR barrier* OR facilitator*) AND (pharmacist* OR community pharmacist*) AND (community OR outpatient OR primary care OR primary healthcare OR community pharmacy)]. A snowballing search strategy was also used to identify articles cited in relevant review studies. Furthermore, manual search was performed in google scholar and relevant pharmacy journals. Pharmacy journals searched were: International Journal of Clinical Pharmacy, International Journal of Pharmacy Practice, Research in Social and Administrative Pharmacy, Journal of Clinical Pharmacy and Therapeutics, Journal of Pharmacy Practice and Research, European Journal of Hospital Pharmacy, Pharmaceutical Journal, Journal of American Pharmacist Association. Journal of Antimicrobial Chemotherapy was also searched as this journal covers AMS relevant articles involving health professionals including pharmacists. SKS and SP independently selected studies after the screening of titles and abstracts followed by reviewing full-text using the Covidence [25] platform. Papers were excluded if it was clear from the title or abstract that study did not meet inclusion criteria as stated below. Discrepancies were resolved by discussion between SKS and SP.

### 2.3. Study Selection 

#### 2.3.1. Inclusion Criteria

1 A national or cross-sectional survey that explored AMS at community pharmacy context.
2 Survey participants were limited to CPs of any age and level of experiences.
3 Surveys that employed a single or multiple outcome measure related to CPs’ knowledge, perceptions, practices, barriers and facilitators concerning AMS.
4 Full text articles are available.

#### 2.3.2. Exclusion Criteria

1 Qualitative interviews, editorials, reports, case studies and case series;
2 Any survey that did not include AMS as a topic;
3 Study conducted in other than primary care;
4 Articles not written in English language.

### 2.4. Data Extraction

Data were extracted using a customized and constructed data extraction form. We modified the data extraction template used for our previous systematic review [17]. Data extraction template are shown in supplementary Tables S3 and S4. Data were extracted and interpreted by SKS and CB. Data included study demographics, author(s), publication year, place of study, survey design, response rate and outcome measures. An extraction template was generated to describe the survey instruments; validity and reliability of the instrument, sources of survey items, domains of survey constructs, settings and quality.

### 2.5. Assessing the Quality of Included Survey Studies

SKS and CB assessed the quality of surveys as high-, medium- or low- quality using published criteria [26] (Supplementary Table S2). A 10-points scoring scale was applied to determine the high quality (score ≥ 8), medium quality (5 ≤ score < 8) and low quality (score < 5) survey studies.

### 2.6. Collating, Summarising and Analysing Outcome Measures

An evidence synthesis method [27] was used to map the comprehensive evidence of CP-AMS. SKS extracted the evidence on the characteristics of survey tools and the outcomes using the extraction templates. SKS and CB discussed and planned the analysis of evidence. We summarized six outcomes that were reported in ten surveys: knowledge or awareness of AMS, perception about AMS, AMS activities, barriers to implementing AMS, facilitators of implementing AMS and recommendations to address barriers. Meta-analysis was not performed for any of the outcomes assessed because of a lack of consistency in survey items. AMS perceptions and practices were measured by an agreement scale and agreement percentages of respective items. We focused on the outcomes of key AMS practices by CPs that were highlighted by the WHO [9].

Synthesis of quantitative data was based on grouping similar fixed responses into categories. The percentage median and inter-quartile range (IQR) were determined where two or more responses were available in a fixed response category. Categories were grouped into perceived knowledge of AMS; perceptions of AMS and AMS practices. A socioecological framework [28] was used as a guide to classify the barriers and facilitators perceived by CPs to undertake AMS at five different levels: personal, interpersonal, community/policy, health system structure and financial level. Intervention recommendations to address the barriers to driving AMS were described.

## 3. Results

### 3.1. Breadth of Survey Studies

**Figure 1:**
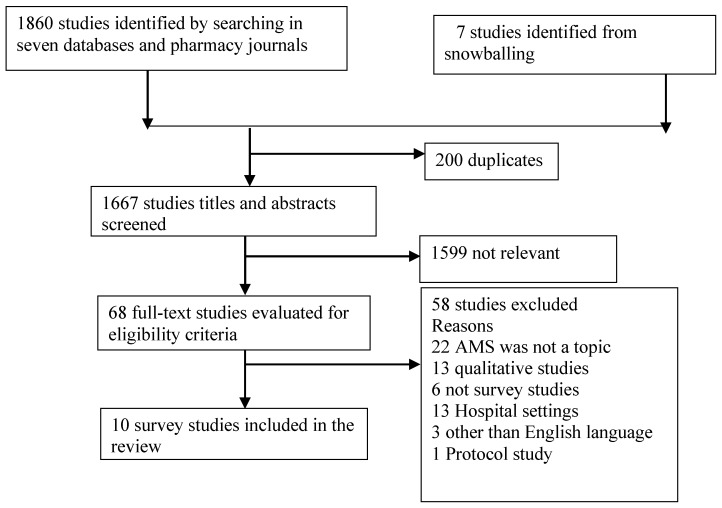
Selection of study.

We identified 1860 articles, of which 68 were full text reviewed after the screening of titles and abstracts. Ten surveys [29,30,31,32,33,34,35,36,37,38] were included in this review (Figure 1).

### 3.2. Study Demographics and Description of Survey Tools

Table 1 describes the characteristics of survey studies and validity of survey tools. Ten studies [29,30,31,32,33,34,35,36,37,38] were conducted in six countries in the years ranging from 2015 to 2018. Survey studies were conducted in: two each from Australia, UK and Pakistan, and one from each of Malaysia, Qatar, Canada, and Ethiopia. Seven surveys were conducted in developed countries and three in low and middle-income countries.

All ten studies were cross-sectional surveys. The surveys were administered either online (*n* = 2) or paper-based (*n* = 8). Ten surveys included responses for a total of 1530 CPs. The response rate of survey studies varied from 12.4–96.6%. Two studies did not report the response rate. Five studies used validated instruments; three of five studies used the same validated instrument. Five studies developed a self-administered questionnaire based on literature reviews but did not report validation studies of the tool except one. Five studies used Cronbach alpha to assess the internal consistency of the questionnaires. Only one survey used exploratory factor analysis (EFA) to evaluate the internal structure and construct validity. Survey data were analysed using descriptive statistics (*n* = 10), linear regression model (*n* = 3), univariable and multivariable regression model (*n* = 2), exploratory factor analysis (*n* = 1) and inferential statistics (*n* = 4).

### 3.3. Quality Assessment

According to the quality assessment of the surveys using predefined published criteria [26] four studies were found as the high quality, two were of medium quality and four were of low quality (Table 2).

### 3.4. Reported Survey Outcomes

Table 1 presents the outcome domains reported in the survey studies. No surveys assessed the AMS knowledge using knowledge measurement tools or construct. Two studies measured perceived knowledge about AMS. Seven studies assessed AMS perceptions or attitudes and eight measured AMS practices. Five studies reported barriers to conducting AMS and four studies reported the facilitators to accelerate AMS. Three studies reported both barriers and facilitators to undertake AMS by CPs.

#### 3.4.1. Knowledge about AMS

No studies used any metrics to measure the knowledge of AMS. Perceived knowledge construct of AMS was reported in terms of CPs’ familiarity or hearing of the term of AMS. Most CPs were familiar with the term of AMS. According to an included study [29], 75% of CPs’ understanding of AMS improved after their reading of the definition of AMS as defined by the Infectious Disease Society of America (IDSA).

#### 3.4.2. Perceptions of AMS

CPs’ perceptions of AMS are summarized in Table 3. Seven of ten surveys found a positive perception of CPs towards the importance of AMS. Though perceptions varied with experience and educational qualification of CPs. Two surveys showed that CPs’ work experiences and post-graduate qualifications, both were associated with positive perceptions towards AMS. Most CPs believed that AMS improved patient care and reduced inappropriate use of antibiotics. Higher proportions of CPs believed that they had a positive role in AMS and were willing to participate in future initiatives and educational programs related to AMS. Most CPs also felt that health professionals other than prescribers were needed to understand AMS. However, approximately half of the CPs perceived that their individual efforts on AMS might have minimal impact on reducing resistance in the community.

#### 3.4.3. AMS Practices

The self-reported AMS practices of CPs were evaluated. AMS practice items were grouped into four categories: collaboration with prescribers; educating patients; antimicrobial dispensing process; and participation in AMS campaign. AMS practices are summarized in Table 4 and described below.

##### Communication with the Prescribers

Most CPs performed communication with prescribers when there was uncertainty in appropriateness of antibiotic prescriptions including when the patient was allergic to the prescribed antibiotic. Participant CPs contacted prescribers when they thought that the choice of antibiotic was not optimal and often communicated with other health professionals pertaining to infection control and AMS. Most CPs referred their patients to GPs when symptoms were suggestive of an infection and felt that their collaboration with GPs were restricted by a lack of collaborative system structure.

##### Patient Education

Less than half of CPs educated patients on the use of antimicrobials and antimicrobial resistance. About half of the CPs provided antibiotic use information to their patients. In contrast, most CPs gave advice when it would be appropriate to use repeat antibiotic prescriptions and provided a clear message on the expected side effects of using prescribed antibiotics.

##### AMS Compliant Dispensing Process

One-third of CPs screened guideline-compliance of antimicrobial prescription. Similar proportions of CPs evaluated prescription according to good dispensing practice guidelines. In contrast, most of the time CPs considered clinical safety parameters such as drug interaction, allergy and adverse drug reactions before dispensing antimicrobials. CPs were generally supportive of dispensing no antibiotics for “delayed antibiotic prescriptions” within 24 h of seeing doctors. CPs preferred recommending over the counter (OTC)/self-care treatment for an infection where antibiotics were not needed. Most often CPs dispensed antimicrobials with more than the prescribed duration of antibiotics on the patient’s request in some settings.

##### Participation in AMS Campaign

A lower proportion of CPs took part in AMS campaign or AMS awareness initiatives.

#### 3.4.4. Barriers and Facilitators to Implementing AMS

Reported barriers and facilitators that influenced CPs to conduct AMS are summarised in Table 5.

##### Barriers to Undertaking AMS

Major barriers with which the highest proportion of CPs agreed were: lack of training to undertake AMS, access to patient’s records and laboratory data, non-receptive behaviours of GPs to CPs’ intervention on the choice of antibiotics, availability of AMS supportive dispensing guidelines, systems that support interacting with GPs, time-poor settings, remunerations and reimbursement models. Furthermore, CPs believed that their undefined roles in AMS and patients’ lack of understanding of their role in AMS were important barriers to implement AMS in community pharmacy.

##### Facilitators of Conducting AMS

Reported facilitators to improve AMS practices by CPs included their familiarity with AMS, willingness to participate AMS programs, positive intention to collaborate with GPs, public awareness campaign, accessibility to antibiotic guidelines, and patients’ clinical and laboratory reports. Most CPs believed that the provision of AMS education, defining their roles in AMS and financial incentives l would facilitate AMS activities in community pharmacy.

## 4. Discussion

There exists a small, but growing body of literature concerning CP-AMS. Ten surveys were found from seven countries globally that measured perceptions and practices of CPs in relation to AMS. The dearth of survey studies may be due to suboptimal progress of AMS implementation in the community compared to hospitals settings. Only three validated surveys were found to assess CPs’ perceptions and practices but no validated survey assessed AMS knowledge. Local validation of these survey tools is required to ensure their suitability and validity within local contexts.

The existing AMS surveys are suboptimal to precisely monitor and evaluate the progress of AMS in community pharmacy in line with the country specific national AMR action plan. Some stewardship components could be included to optimally design AMS surveys in future. Therefore, there is much room for improvement in the design of surveys and survey questions to investigate the availability of AMS implementation resources, guidelines, interprofessional collaborative structures and staff required to perform AMS by CPs. Questions related to the changes in infrastructures, organizational setup and pharmacy owners’ attitudes and motivations to support AMS programs could also be included. Additionally, CPs’ attitudes towards GP-pharmacist collaboration strategies, and attitudes towards the feasibility of evidence-based AMS strategies in the context where CPs work should be explored to optimally design AMS programs.

Though most CPs knew the term of AMS, gaps in their AMS activities were found in this review. CPs had little understanding of what they are supposed to do when dispensing antibiotics, managing infections and providing patient care that is guided by AMS principles. WHO also reported that the roles of CPs are not well defined in policy documents of most countries [9]. Surveyed CPs felt that their roles need to be defined. In doing so, stakeholders and policymakers should assess and consider CPs’ positive attitudes to facilitate AMS and promote their roles for patient safety.

This review demonstrated that nearly half (53.0 %, IQR, 43.2–67.4 %) of CPs educated patients about the use of antibiotics and resistance. Exploration is required on how to make sure the availability of patient’s leaflets and guidelines that can be used by CPs to provide patients the self-care advice for infections, antibiotic compliance advice, and the message that inappropriate antibiotics can kill the protective bacteria in the gut, develop diarrhoea and produce secondary infections [12].

Klepser et al. highlighted that a strong GP-CP collaboration is a fundamental strategy to develop sustainable stewardship interventions in the community [39]. The lack of GP-CP communication hindered CPs to provide interventions on antibiotic prescriptions. CPs felt uncomfortable with the idea about the monitoring and evaluation of antibiotic prescriptions prescribed by GPs. Two studies [31,32] of this review explained this uncomfortable attitude by the lack of their awareness about local antibiotic guidelines, and anticipated impact on relationship with prescribers. Furthermore, lack of confidence of CPs and GPs’ belief that monitoring prescriptions is not a part of CPs’ roles were also reported as barriers for CPs to comfortably judge GPs’ antibiotic prescriptions. This prescription judgement role made CPs themselves feel intruded upon and also this role was unappreciated by GPs that is comparable with an American study [16]. It is, therefore, worthwhile to assess the attitudes of both GPs and CPs towards GP-CP collaboration in AMS in different country contexts in future.

Two studies in Saudi Arabia [40] and China [41] demonstrated the practice of dispensing antibiotics without prescriptions by most CPs that is associated with the overuse of antibiotics and the risk of developing AMR. Such practices have been aggravated by financial incentives [42,43]. The economic interventions that may change CPs’ attitudes to avoid dispensing antibiotics without a prescription could be investigated.

The longer duration of antibiotic therapy has been reported as a promoter of the development of AMR by two studies [44,45]. Approximately 30% of CPs often or always dispensed antibiotics for longer durations than prescribed by physicians in a survey in Pakistan [36]. Therefore, context-dependent significant attempts are required to develop AMS friendly dispensing guidelines and a strict policy to reduce the dispensing of prolonged duration antibiotics that deviates from doctors’ prescriptions. CPs’ barriers to engage in AMS was also involved at the financial level; no monetary compensation for saving inappropriate antibiotic use and spending extra time for giving AMS services. Feasible incentives mechanism for CPs to conduct AMS activities in community pharmacy could be worthy of future investigation.

The context-specific understanding of CPs’ knowledge, perceptions, practices and barriers related to AMS has unique importance to develop and facilitate CP-led AMS interventions. Jamshed et al. highlighted in his review that majority of CPs are still selling antibiotics for unjustified reasons although they have good awareness and knowledge of antibiotic dispensing in low- and middle-income country settings [46]. In this regard, development of AMS survey tools at local, national and international contexts could be an important AMS resource. And conducting AMS surveys using these tools in years ahead would help to monitor and evaluate gaps and signs of progress of AMS in community pharmacy in order to inform developing the feasible CP-AMS strategies.

This review had some strengths. First, this is the first scoping review which looked at AMS survey of CPs. Second, PRISMA-ScR checklists and a methodological framework ensured the best practice of conducting this review. Third, quality assessment added methodological updates to develop future CP-AMS survey instruments. Fourth, interpretations of quantitative and qualitative data added a bit more understanding of perceptions, practices and barriers to implementing AMS by CPs.

This review had some limitations. First, outcomes were based on a limited number of surveys. Second, while interpreting evidence, it faced some methodological limitations: unclear validity of questions posed; variation in sample size and response rate; and non-response bias. The findings may thus overestimate the AMS perceptions and practices of CPs. Third, literature was limited to only English language. Fourth, differences in pharmaceutical legislation and laws of countries where surveys were performed may influence AMS activities of pharmacists.

## 5. Conclusions

There exists a small but growing body of AMS survey instruments that can be used for exploring CP-AMS. The validity of CP-AMS survey tools at local, national and international contexts requires to be established. There is a room for improving the standard of existing AMS survey tools with evidence-based AMS strategies, stewardship targets, key stewardship components and attitudes towards prescriber-pharmacist collaboration to identify stewardship needs and monitor stewardship progress in community pharmacy. Contextual variations in pharmacy practices prevent us from drawing firm conclusions on the knowledge, perceptions and practices of CPs regarding AMS at this point. With the limited evidence, CPs positively perceived AMS but felt that their AMS practice improvements require AMS training and guidelines, a GP-CP collaborative system structure, and defining their roles to perform AMS. Further surveys could also be designed to gain better qualitative insights into the barriers at individual, practice, system and policy level to conducting AMS by CPs. This review recommends researching the mechanism on how to enhance the engagement of CPs in AMS and develop a GP-CP collaborative AMS implementation model to address AMR in primary care.

## Figures and Tables

**Table 1:** Characteristics and validity of survey studies.

Study Author Year	Country and Population	Methods and Mode	Response Rate	Questionnaire Developed by	Validation and no. of Questions	Outcome Domain	Reliability	Quality
Rizvi et al., 2018	Australia Tasmanian CPs	Cross-sectional survey Online Email, fax and post	61% (85/140)	Rizvi et al.	Validated 38	K, Per, P, B, F	+	High
Khan et al., 2016	Malaysia CPs from State of Selangor	Cross-sectional survey Paper-based	83.5% (188/225)	Khan et al.	Validated 24	Per, P	+	High
Erku et al., 2016	Ethiopia CPs from eight cities of Ethiopia	Cross-sectional survey Paper-based	86.6% (334/389)	Khan et al.	Validated 24	Per, P	+	High
Pawluk et al., 2015	Qatar CPs from Qatar	Cross-sectional survey Paper-based workshop	51.6% (32/62)	Pawluk et al.	Developed by literature review 13	Per, F	-	Low
Avent et al., 2018	Australia CPs from Queensland	Cross-sectional survey Online e-newsletter	- 120 responses	Avent et al.	Not validated 21	P, B	-	Low
Sarwar et al., 2018	Pakistan CPs from Punjab province	Cross-sectional survey paper-based	96.6% (400/441)	Sarwar et al. and Khan et al.	Validated 29	A, P, B, F	+	High
Wilcock et al., 2017	UK	Cross-sectional survey Paper-based	91.9% (57/62)	Wilcock et al.	Not validated 10	P, B	-	Low
Rehman et al., 2018	Pakistan Urban settings	Cross-sectional survey Paper-based	37% (20/67)	Khan et al.	Validated 26	Per, P	+	Medium
Hancock et al., 2016	UK CPs of Huddersfield town centre	cross sectional survey Paper-based	- 50 respondents	Hancock et al.	Not validated 28	A, P, B, F	-	Medium
Lee et al., 2017	Canada CPs from Saskatchewan	Cross sectional survey Paper-based	12.4% (138/1109)	Lee et al.	Not validated 19	K, A	-	Low

K = knowledge, A = attitudes, Per = perception, P = practice, B = barriers, F = facilitators, Reliability (Cronbach alfa): measured (+), not measured (−).

**Table 2:** Quality assessment of survey studies.

N	Criteria	Reviewer (R)	Rizvi et al., 2018	Khan et al., 2016	Erku et al., 2016	Pawluk et al., 2015	Avent et al., 2018	Sarwar et al., 2018	Wilcock et al., 2017	Rehman et al., 2018	Hancock et al., 2016	Lee et al., 2017
1	Was there a clearly defined research question?	R1	✓	✓	✓	✓	✓	✓	✓	✓	✓	✓
		R2	✓	✓	✓	✓	✓	✓	✓	✓	✓	✓
2	Did the authors select samples that well represent the population to be studied?	R1	✓	✓	✓	x	x	✓	x	x	✓	x
		R2	✓	✓	✓	x	?	✓	x	x	✓	x
3	Did the authors use designs that balance costs with errors?	R1	?	x	?	x	?	?	x	?	?	?
		R2	?	x	x	x	?	?	x	?	?	?
4	Did the authors describe the research instrument?	R1	✓	✓	✓	x	x	✓	x	✓	✓	✓
		R2	✓	✓	✓	?	x	✓	x	✓	✓	?
5	Was the instrument pretested?	R1	✓	✓	✓	✓	x	✓	x	✓	?	x
		R2	✓	✓	✓	✓	x	✓	?	✓	?	?
6	Were quality control measures described?	R1	✓	?	✓	x	?	✓	x	✓	✓	x
		R2	✓	?	✓	x	x	✓	x	?	✓	?
7	Was the response rate sufficient to enable generalizing the results to the target population?	R1	✓	✓	✓	x	?	✓	x	x	x	x
		R2	✓	✓	✓	x	x	✓	x	x	x	x
8	Were the statistical, analytic, and reporting techniques appropriate to the data collected?	R1	✓	✓	✓	✓	✓	✓	?	x	✓	✓
		R2	✓	✓	✓	✓	✓	✓	x	x	?	✓
9	Was evidence of ethical treatment of human subjects provided?	R1	✓	✓	✓	✓	✓	✓	✓	✓	✓	✓
		R2	✓	✓	✓	✓	✓	✓	✓	✓	✓	✓
10	Were the authors transparent to ensure evaluation and replication?	R1	✓	✓	✓	x	x	✓	x	✓	x	x
		R2	✓	✓	?	x	x	✓	x	✓	?	x
Quality of survey studies			H	H	H	L	L	H	L	M	M	L

H = high quality; M = medium quality; L = low quality; ? = unclear; x = no; ✓ = yes. Scoring: high quality (score ≥ 8), medium quality (5 ≤ score < 8) and low quality (score < 5).

**Table 3:** Perceptions of community pharmacists (CPs) towards antimicrobial stewardship (AMS).

Items	Median (%)	IQR
AMS improve patient care (*n* = 6)	86.0	83.3–93.5
AMS reduce inappropriate use (*n* = 2)	84.0	83–85
CPs have important role in AMS (*n* = 4)	93.0	90.8–94.7
Willing to participate in future AMS initiatives (*n* = 6)	87.8	83.6–90.3
AMS should be practiced at community pharmacy level (*n* = 3)	78.0	52.5–79.3
AMS reduce infection associated costs (*n* = 1)	78.0	–
Health-care professionals other than prescribers need to understand AMS (*n* = 3)	69.0	66.8–84.5
Individual efforts at AMS have minimal impact on the antimicrobial resistance problem (*n* = 3)	51.4	40.7–69.4

**Table 4:** AMS practices of CPs.

AMS Practice Components	% CPs Often or Always Do This Practice	
	Median	IQR
**Collaboration with prescribers**		
Collaborate with prescribers in case of uncertainty in appropriateness of antibiotic prescription (*n* = 5)	77.0	55.2–77.8
Collaborate with other health care professionals for infection control and AMS (*n* = 4)	54.7	34.8–63.2
Contacting prescriber when patient is allergic to prescribed antibiotic (*n* = 1)	98.6	–
Contacting prescriber when choice of antibiotic may not be optimal (*n* = 1)	46.5	–
**Educating patients**		
Provide antibiotic information to patients (*n* = 1)	56	–
Educate patients on the use of antimicrobials and drug resistance issues (*n* = 5)	53.0	43.2–67.4
Provide clear message on expected side effect of using antibiotics (*n* = 1)	86	–
Provide advice to the patients when it would be appropriate to use repeat (*n* = 1)	82.9	–
**Dispensing process**		–
Dispense antimicrobials without prescription (*n* = 5)	34.1	19.4–47.0
Screen antimicrobial prescription in accordance with guidelines before dispensing (*n* = 3)	47.5	25.2–58.3
Consider clinical safety parameters (drug interaction, allergy, ADRs) before dispensing (*n* = 5)	68.7	53.6–70.7
Evaluate prescription according to good dispensing practice guidelines (*n* = 1)	33.4	–
Refer patients to general practitioners when symptoms are suggestive of an infection (*n* = 1)	99	–
Recommending over the counter (OTC)/self-care treatment to patient with infections not needing antibiotics (*n* = 1)	95.8	–
Do not dispense delayed antibiotic prescription within 24 h of seeing doctor (*n* = 1)	60	–
Dispensed antibiotics for longer durations than prescribed by physicians (*n* = 2)	18.4	13.6–23.2
**Participation in AMS campaign**		
Take part in AMS campaign/awareness movement (*n* = 1)	40.9	20.4–41.5

**Table 5:** Barriers to and facilitators in implementing AMS by CPs.

Barriers	Facilitators	Proposed Recommendation to Improve AMS in Community Pharmacy
**Personal**	**Personal**	**Personal level**
Education and training	Familiarity of AMS term Positive perception about AMS Willingness to participate future AMS training, workshop or conferences Skills of assessing drug interaction, adverse drug reactions (ADRs) and allergies to prescribed antibiotics	Provision of AMS training as a part of the CPD program
**Interpersonal**	**Interpersonal**	**Interpersonal-level**
Prescriber-CP interaction Non-receptive behaviours of GPs to pharmacist intervening the choice of antibiotics Fear of losing relationship with GPs while measuring guideline compliance of antimicrobial prescriptions	Positive intention to collaborate with prescribers	GP-CP network (policy guided) Local GP-pharmacy practice agreement
**Community/policy**	**Community/policy**	**Community/policy level**
provision of AMS campaign prolonged (e.g.,12 months) repeat dispensing of antibiotic policy Culture of GP-pharmacy team-based service CPs’ roles are not defined in AMS Limited patient awareness about CPs’ role in AMS	Professional organisation’s training modules and tool kits (e.g., NPS Medicine Wise, CDC, NHS)	Restriction on OTC sale of antibiotics Provision of providing audit and feedback data on both prescribing and dispensing Provision of patient education on antibiotic use, resistance and repeat use Use of patients leaflets (e.g., self-care advice for infections and antibiotic compliance advice) Public awareness campaign relevant with AMS Pharmacy professional organizations should define the role of CPs in AMS as a policy document
**Health system structure**	**Health system structure**	**Health system structure-level**
Accessibility of patient’s records and laboratory data No AMS compliant dispensing guidelines Technology that supports GP-CP communication Time poor settings No provision of point-of-care (POC) testing service to differentiate bacterial or viral infection	-	Decision support tools (antimicrobials review tools) IT technology Provision of guidelines to undertake AMS in pharmacy practices POC testing services and relevant training for CPs Provision of use of therapeutic guidelines by CPs to ensure appropriateness of antimicrobials
**Financial**	**Financial**	**Financial-level**
Reimbursement models Remuneration for AMS services		Remuneration for pharmacies involved in AMS programs Financing mechanism for GP-pharmacy collaboration

## Supplementary Materials

The following are available online at https://www.mdpi.com/2079-6382/8/4/263/s1. Table S1: Preferred reporting items for systematic reviews and meta-analyses extension for scoping reviews (PRISMA-ScR) checklist. Table S2: Quality reporting criteria used to assess the quality of survey studies. Table S3: Data extraction template for study characteristic and quantitative outcomes. Table S4: Data extraction template for study characteristic and qualitative outcomes on barriers and facilitators to improve AMS.
