# Supplementary material for: Knowledge, Perceptions and Practices of Community Pharmacists Towards Antimicrobial Stewardship: A Systematic Scoping Review
Source: Antibiotics (Basel). 2019 Dec 12;8(4):263. doi: 10.3390/antibiotics8040263 (PMC6963969; doi:10.3390/antibiotics8040263)
Supplement: Supplementary file 1 [file antibiotics-08-00263-s001.zip › Supplementary_material.docx]

**Supplementary Material**

**Supplementary Table S1: Preferred Reporting Items for Systematic reviews and Meta-Analyses extension for Scoping Reviews (PRISMA-ScR) Checklist^24^**

| **SECTION** | **ITEM** | **PRISMA-ScR CHECKLIST ITEM** | **REPORTED ON PAGE #** |
| --- | --- | --- | --- |
| **TITLE** | | | |
| Title | 1 | Identify the report as a scoping review. | 1 |
| **ABSTRACT** | | | |
| Structured summary | 2 | Provide a structured summary that includes (as applicable): background, objectives, eligibility criteria, sources of evidence, charting methods, results, and conclusions that relate to the review questions and objectives. | 1 |
| **INTRODUCTION** | | | |
| Rationale | 3 | Describe the rationale for the review in the context of what is already known. Explain why the review questions/objectives lend themselves to a scoping review approach. | 2,3 |
| Objectives | 4 | Provide an explicit statement of the questions and objectives being addressed with reference to their key elements (e.g., population or participants, concepts, and context) or other relevant key elements used to conceptualize the review questions and/or objectives. | 3 |
| **METHODS** | | | |
| Protocol and registration | 5 | Indicate whether a review protocol exists; state if and where it can be accessed (e.g., a Web address); and if available, provide registration information, including the registration number. | N/A |
| Eligibility criteria | 6 | Specify characteristics of the sources of evidence used as eligibility criteria (e.g., years considered, language, and publication status), and provide a rationale. | 4 |
| Information sources* | 7 | Describe all information sources in the search (e.g., databases with dates of coverage and contact with authors to identify additional sources), as well as the date the most recent search was executed. | 3 |
| Search | 8 | Present the full electronic search strategy for at least 1 database, including any limits used, such that it could be repeated. | 3,4 |
| Selection of sources of evidence† | 9 | State the process for selecting sources of evidence (i.e., screening and eligibility) included in the scoping review. | 4 |
| Data charting process‡ | 10 | Describe the methods of charting data from the included sources of evidence (e.g., calibrated forms or forms that have been tested by the team before their use, and whether data charting was done independently or in duplicate) and any processes for obtaining and confirming data from investigators. | 4,5 |
| Data items | 11 | List and define all variables for which data were sought and any assumptions and simplifications made. | 4,5 |
| Critical appraisal of individual sources of evidence§ | 12 | If done, provide a rationale for conducting a critical appraisal of included sources of evidence; describe the methods used and how this information was used in any data synthesis (if appropriate). | N/A |
| Synthesis of results | 13 | Describe the methods of handling and summarizing the data that were charted. | 5 |
| **RESULTS** | | | |
| Selection of sources of evidence | 14 | Give numbers of sources of evidence screened, assessed for eligibility, and included in the review, with reasons for exclusions at each stage, ideally using a flow diagram. | 5 |
| Characteristics of sources of evidence | 15 | For each source of evidence, present characteristics for which data were charted and provide the citations. | 5,6 |
| Critical appraisal within sources of evidence | 16 | If done, present data on critical appraisal of included sources of evidence (see item 12). | 6,17 |
| Results of individual sources of evidence | 17 | For each included source of evidence, present the relevant data that were charted that relate to the review questions and objectives. | 15, 16 |
| Synthesis of results | 18 | Summarize and/or present the charting results as they relate to the review questions and objectives. | 6-8, 18-21 |
| **DISCUSSION** | | | |
| Summary of evidence | 19 | Summarize the main results (including an overview of concepts, themes, and types of evidence available), link to the review questions and objectives, and consider the relevance to key groups. | 9-10 |
| Limitations | 20 | Discuss the limitations of the scoping review process. | 10 |
| Conclusions | 21 | Provide a general interpretation of the results with respect to the review questions and objectives, as well as potential implications and/or next steps. | 11 |
| **FUNDING** | | | |
| Funding | 22 | Describe sources of funding for the included sources of evidence, as well as sources of funding for the scoping review. Describe the role of the funders of the scoping review. | 12 |

**Supplementary Table S2: Quality reporting criteria used to assess the quality of survey studies**

| 1. Was there a clearly defined research question? |
| --- |
| a. Are the study objectives clearly identified? |
| b. Did the authors consider alternatives to using a survey technique to collect information? (ie, did they justify using survey research methods?) |
| — AACP databases |
| — Readily available literature |
| — Other professional organizations |
| 2. Did the authors select samples that well represent the population to be studied? |
| a. What sampling approaches were used? |
| b. Did the authors provide a description of how coverage and sampling error were minimized? |
| c. Did the authors describe the process to estimate the necessary sample size? |
| 3. Did the authors use designs that balance costs with errors? (e. g, strive for a census with inadequate follow-up versus smaller sample but aggressive follow-up) |
| 4. Did the authors describe the research instrument? |
| a. Was evidence provided regarding the reliability and validity of an existing instrument? |
| b. How was a new instrument developed and assessed for reliability and validity? |
| c. Was the scoring scheme for the instrument sufficiently described? |
| 5. Was the instrument pretested? |
| a. Was the procedure used to pre-test the instrument described? |
| 6. Were quality control measures described? |
| a. Was a code book used? |
| b. Did the authors discuss what techniques were used for verifying data entry? |
| 7. Was the response rate sufficient to enable generalizing the results to the target population? |
| a. What was the response rate? |
| b. How was response rate calculated? |
| c. Were follow-ups planned for and used? |
| d. Do authors address potential nonresponse bias? |
| 8. Were the statistical, analytic, and reporting techniques appropriate to the data collected? |
| 9. Was evidence of ethical treatment of human subjects provided? |
| a. Did the authors list which IRB they received approval from? |
| b. Did the authors explain how consent was obtained? |
| 10. Were the authors transparent to ensure evaluation and replication? |
| a. Was evidence for validity provided? |
| b. Was evidence of reliability provided? |
| c. Were results generalizable? |
| d. Is replication possible given information provided? |

**Supplementary Table S3: Data extraction template for study characteristic and quantitative outcomes**

| **Study Author, Year** | **Country and**  **Population** | **Methods,**  **Design and**  **validation** | **Response rate**  **No. of survey items** | **Analysis** | **AMS**  **Knowledge** | **AMS**  **Perceptions** | **AMS Practices** | | | | | | | |
| --- | --- | --- | --- | --- | --- | --- | --- | --- | --- | --- | --- | --- | --- | --- |
|  |  |  |  |  |  |  | **Collaboration with prescriber** | **Patient**  **education** | **Monitoring prescription** | **Dispensing** | **Delayed/repeat prescription** | **guidelines** | **Participation in AMS programs** | **Other** |
| Study 1 |  |  |  |  |  |  |  |  |  |  |  |  |  |  |
| Study 2 |  |  |  |  |  |  |  |  |  |  |  |  |  |  |
| Study 3 |  |  |  |  |  |  |  |  |  |  |  |  |  |  |
| Study 4 |  |  |  |  |  |  |  |  |  |  |  |  |  |  |
| Study 5 |  |  |  |  |  |  |  |  |  |  |  |  |  |  |
| Study 6 |  |  |  |  |  |  |  |  |  |  |  |  |  |  |
| Study 7 |  |  |  |  |  |  |  |  |  |  |  |  |  |  |
| Study 8 |  |  |  |  |  |  |  |  |  |  |  |  |  |  |
| Study 9 |  |  |  |  |  |  |  |  |  |  |  |  |  |  |
| Study 10 |  |  |  |  |  |  |  |  |  |  |  |  |  |  |

**Supplementary Table S4: Data extraction template for study characteristic and qualitative outcomes on barriers and facilitators to improve AMS**

| **Study Author, Year** | **Place and**  **Design** | **Methods** | **Response rate** | **Analysis** | **Barriers** | **Facilitators** | **Proposed intervention/recommendations** |
| --- | --- | --- | --- | --- | --- | --- | --- |
| Study 1 |  |  |  |  |  |  |  |
| Study 2 |  |  |  |  |  |  |  |
| Study 3 |  |  |  |  |  |  |  |
| Study 4 |  |  |  |  |  |  |  |
| Study 5 |  |  |  |  |  |  |  |
| Study 6 |  |  |  |  |  |  |  |
| Study 7 |  |  |  |  |  |  |  |
| Study 8 |  |  |  |  |  |  |  |
| Study 9 |  |  |  |  |  |  |  |
| Study 10 |  |  |  |  |  |  |  |
